# Measuring semantic memory using associative and dissociative retrieval tasks

**DOI:** 10.1098/rsos.231208

**Published:** 2024-02-07

**Authors:** Martin Marko, Drahomír Michalko, Adam Kubinec, Igor Riečanský

**Affiliations:** ^1^ Department of Behavioural Neuroscience, Centre of Experimental Medicine, Slovak Academy of Sciences, Sienkiewiczova 1, Bratislava, 813 71, Slovakia; ^2^ Department of Applied Informatics, Faculty of Mathematics, Physics and Informatics, Comenius University in Bratislava, Mlynská dolina F1, Bratislava, 842 48, Slovakia; ^3^ Department of Psychiatry, Faculty of Medicine, Slovak Medical University in Bratislava, Limbova 12, Bratislava, 833 03, Slovakia

**Keywords:** semantic memory, inhibition, verbal fluency, working memory, psychological assessment

## Abstract

Recent theoretical advances highlighted the need for novel means of assessing semantic cognition. Here, we introduce the associative-dissociative retrieval task (ADT), positing a novel way to test inhibitory control over semantic memory retrieval by contrasting the efficacy of associative (automatic) and dissociative (controlled) retrieval on a standard set of verbal stimuli. All ADT measures achieved excellent reliability, homogeneity, and short-term temporal stability. Moreover, in-depth stimulus level analyses showed that the associative retrieval is easier for words evoking few but strong associates, yet such propensity hampers the inhibition. Finally, we provided critical support for the construct validity of the ADT measures, demonstrating reliable correlations with domain-specific measures of semantic memory functioning (semantic fluency and associative combination) but negligible correlations with domain-general capacities (processing speed and working memory). Together, we show that ADT provides simple yet potent and psychometrically sound measures of semantic memory retrieval and offers noteworthy advantages over the currently available assessment methods.

## 1. Introduction

Semantic cognition is a complex system that underpins the encoding, organization, and retrieval of lexical-semantic representations and knowledge (facts, concepts and their relationships) stored in long-term memory (i.e. ‘semantic memory’ [1]). The concept of semantic memory has evolved remarkably since Tulving's seminal proposals (for a recent review, see [2]), catalysed recently by the advent of cognitive network theory [3,4] and modern neuroscience [5,6]. These converging approaches have grounded the contemporary views that semantic cognition relies on two functionally interacting neurocognitive systems [7,8]. First is the system for *semantic representation*, which encodes and integrates knowledge (conceptual representations) distilled through experience and drives the *automatic retrieval* of information from semantic memory, i.e. a bottom-up activation of semantic representations triggered by environmental cues or spontaneous thought [9,10]. Second is the system for *semantic control*, which regulates the activations within the representational system and so implements the *executive control* of memory search and retrieval, i.e. a top-down manipulation of semantic processing employed whenever the outputs from automatic retrieval are inappropriate for the current task [11]. This framework extends the narrow conceptualization of semantic system as a memory store, highlighting the need to address and understand the mechanisms supporting the information retrieval. Importantly, both the system for lexical-semantic representation as well as the processes that operate upon these representations are fundamentally involved in the regulation of mental activity and shape adaptive behaviours [12,13]. This is evident in individuals with various neuropsychiatric conditions, where the deterioration of the systems leads to profound memory deficits, impaired thinking, socio-affective disturbances, and hindered language abilities (e.g. degradation of knowledge in semantic dementia versus impaired retrieval control in semantic aphasia [14–16]). Consequently, these principal roles of semantic cognition in everyday functioning warrant novel behavioural assessment tools that regard both the recent advances in theory as well as rigorous psychometric criteria for basic research or clinical application. Following that, here we introduce the *associative–dissociative retrieval task* (ADT), a novel method developed to assess automatic (free-associative) and controlled (dissociative) aspects of semantic memory retrieval. Crucially, in addition to these retrieval measures, the ADT allows for the assessment of individuals' inhibitory capacities that support goal-oriented memory retrieval and lexical-semantic processing. Moreover, the use of this novel measure is further promoted by a broad scope of mental faculties in which a role of inhibitory control has been implied and the limited number of currently available assessment tools probing such inhibitory capacities in memory domain.

### 1.1. The associative-dissociative retrieval task

In its essence, the ADT is based on a simple word-associative principle, requiring individuals to generate verbal responses to sequentially presented word stimuli following two specific retrieval rules (conditions). In the *free-associative* condition (FA), participants are instructed to produce the first word that freely comes to mind as related to a presented stimulus. This type of retrieval tasks has a long history in cognitive and clinical research [17,18]. Given its unconstrained nature, producing free word associations is considered a relatively pure measure of automatic memory search and retrieval that imposes little demands on cognitive control [9,10,19,20] but relies on spontaneous activation along memory representations, which is supported primarily by the activity of the default mode network, especially within temporal brain regions [13,20–22]. The classical view on this automatic retrieval process is that the presentation of a cue leads to activation of the relevant parts (units or features) in semantic memory which spreads towards a set of related concepts in the lexical-semantic network, rendering them more accessible for subsequent retrieval [23,24]. This implies that the dynamics and contents of free associative retrieval are implicitly shaped by the pre-established (i.e. habitual) connectivity patterns encoded within the representational system. Support for this implication has also come from recent studies using a network science approach to demonstrate that differences in the connectivity of individuals’ lexical-semantic networks affect the speed and flexibility of memory retrieval as well as high-order cognition [4,25–28]. Taken together, the FA retrieval task represents a simple and straightforward measure to operationalize the automatic memory retrieval that is underpinned by semantic activation spreading and shaped by the intrinsic connectivity of the lexical-semantic networks.

In contrast to FA, the *dissociative* condition (DA) requires participants to generate unrelated words (i.e. dissociates) to serially presented word stimuli. Previous research has shown that retrieving semantically dissociated words exerts substantially more cognitive effort and processing time than the FA task, which has been attributed to additional control demands pertaining to inhibition and response monitoring [29–33]. The putative engagement of the inhibitory mechanisms in the dissociative retrieval stems from the inherent need to suppress the automatic (but task-inappropriate) semantic activations and response candidates evoked by the stimulus words [10,11,34,35]. In line with this account, naming unrelated words recruits the top-down attentional control mechanism, implemented primarily by a prefrontal-parietal brain network, to counteract the stimulus-driven (i.e. bottom-up) activations within the system for semantic representation and so to prevent the triggering of habitual word associates [27,36–38]. These converging lines of evidence allow us to conclude that the DA retrieval task probes the controlled memory retrieval involving the inhibition of prepotent but contextually inappropriate responses.

Moreover, employing the response-time additivity model and subtraction method, the ADT permits estimating the *inhibition cost* (IC), which is calculated as the response latency difference (ΔRT) between the retrieval tasks (IC = DA − FA; for more details, see [31]). This difference measure is based on the assumption that FA and DA involve temporary organized sub-processes (stages) while at least some of these hypothetical stages are shared by the tasks (among other things, both tasks require individuals to read the word, assess its meaning, activate the relevant memory representations, as well as response preparation and its motor execution [10,30]). Therefore, by subtracting the mean retrieval latency of the two retrieval conditions, the processing ‘costs’, response times (RTs) attributable to these shared processes can be effectively cancelled out, and the unique processing time pertaining to the inhibition estimated. Following the rationale, the IC has been proposed to reflect more directly the individual efficiency of inhibition during semantic memory retrieval, which has been supported by the recent experimental and correlational studies [10,27,37,39,40].

Taken together, rooted in the word-associative tradition and recent neurocognitive models [5,8], the ADT provides theoretically sound measures able to probe and disentangle the automatic (free-associative) and controlled (dissociative/inhibitory) processes during semantic memory retrieval.

### 1.2. Goals of the study

The present study provides a psychometric and methodical description of the ADT, developed to assess and differentiate automatic versus controlled semantic memory functions while providing an in-depth analysis of the psychometric properties (reliability and validity) of its main measures and stimulus materials. This report is structured into three parts, which map onto our three major methodological and psychometric goals. The first was to evaluate the internal consistency and test-retest reliability of the main measures (tasks) from the ADT and so to estimate the recommended number of trials (or duration) for each. In the second part, we provide a set of standard materials for the ADT (i.e. word stimuli/items) with specific psycholinguistic and associative properties, and response norms. This part includes a thorough item-level analysis investigating the stimulus properties possibly influencing the retrieval fluency (the primary behavioural outcome), which may help the researchers to tailor the stimulus selection following specific intents. Finally, in the third part of the present study, we further addressed the construct validity of the ADT measures by estimating their correlations with other well-established cognitive measures. For this purpose, we administered additional cognitive tasks assessing processing speed, working memory, associative combination and semantic verbal fluency. These tasks were selected to cover both the domain-specific cognitive capacities that involve semantic processing and retrieval (i.e. semantic verbal fluency and associative combination) as well as domain-general capacities (i.e. processing speed and working memory capacity) not specifically involving such semantic processes but supporting cognitively demanding tasks across multiple domains. Thus, both convergent and discriminant validity was inspected using such measures.

## 2. General methods

We report how we determined our sample size, all data exclusions, all manipulations and all measures in the study. The study was not preregistered.

### 2.1. Participants

A priori power analysis (G*Power 3.1.9 [41]) was used to estimate the sample size. This estimate was based on both the previous studies involving free-associative and dissociative retrieval together with other cognitive measures (*r* ≥ 0.37 in [31,40]) as well as the requirement to detect effect sizes (with power 1 − β = 0.80 at *α* = 0.05) of possible theoretical and/or clinical relevance (using the cut-off criterion suggested by Cohen, i.e. *r* ≥ 0.3 or *d* ≥ 0.5 for simple correlations and paired comparisons, respectively). Following that, a total of 102 young adults (58 females, mean age 22.8 ± 2.75 years) took part in the study. All participants were native Slovak speakers with native-level language proficiency. The individuals were primarily undergraduate students (predominantly from humanities and STEM fields, equally represented) with no history of severe head trauma, previous or current treatment for a neurological or psychiatric condition, or use of psychoactive substances. The sample comprised 82.8% right-handers, 11.1% mixed-handers and 6.1% left-handers as indicated by the short-form Edinburgh handedness inventory [42]. Upon arrival to the laboratory, participants indicated (on a 5-point ordinal scale) their current level of stress, concentration, psychological arousal, self-confidence, motivation to perform, weariness and the quality of last night sleep. Overall, participants reported low levels of stress and weariness (medians = ‘rather low’), high levels of concentration, motivation to perform, psychological arousal and quality of sleep (medians = ‘rather high’), and mediocre self-confidence. Testing procedures were carried out in accordance with the Declaration of Helsinki [43] and approved by the review board of the Institute of Normal and Pathological Physiology at the Slovak Academy of Sciences. All participants gave written consent upon arrival and received financial compensation for their participation.

### 2.2. General procedure

Cognitive performance was assessed individually, in a single session including three counterbalanced testing blocks (A, B and C). Dividing the cognitive assessment into three homogeneous blocks was adopted as optimal after considering the relevant methodological aspects of the task administration (e.g. counterbalancing, carry-over effects), suitable data analytical options (e.g. principal component analyses (PCA)), expected duration of the tasks, number of trials required to achieve adequate levels of measurement precision, as well as the possible effects of fatigue. Each block lasted approximately 30 to 40 min and consisted of five cognitive tasks administered in the following order: (i) processing speed, (ii) ADT, (iii) working memory capacity, (iv) associative combination, and (v) verbal fluency (described in detail below). Thus, three specific tasks (or parallel forms including a unique set of stimuli) were used to indicate these cognitive abilities (theoretical constructs) in the study (table 1). All tasks were administered using a computer in PsychoPy (version 3.2.4) [44]. At the end of the session, all study participants were given the list of all 120 ADT stimulus words and asked to deliver four associations to each, providing us with data to estimate word association norms. These norms were used to calculate associative properties for the stimuli (as further described in part 2). The relevant materials, codes, and data are available at the Open Science Framework (OSF; https://osf.io/z98my/).

**Table 1.** Overview of the cognitive constructs and measures. (Note: the table lists all tasks used to indicate the cognitive constructs (domains) of interest, their length and internal consistency (McDonald's *ω*). Each construct was assessed using three specific tasks (or parallel forms) across three testing blocks (A, B, C), whereas the order of the blocks was counterbalanced. Within each testing block, the cognitive constructs were assessed in a fixed order (starting with a processing speed task and ending with a semantic verbal fluency task).)

construct	block	task/condition	length	*ω*
processing speed	A	digit-symbol substitution	64 trials	0.715
	B	letter matching	60 trials	
	C	choice response time	72 trials	
automatic retrieval	ABC^a^	free-associative task (FA)	40 trials/block	0.941
controlled retrieval	ABC^a^	dissociative task (DA)	40 trials/block	0.942
inhibition (cost)	ABC^a^	RT difference (DA-FA)	40 trials/block	0.908
working memory capacity	A	alpha span	until 5 errors	0.763
	B	operation span	until 5 errors	
	C	rotation span	until 5 errors	
associative combination	ABC^a^	associative combination task	22 trials/block	0.866
semantic verbal fluency	A	animals	90 s	0.750
	B	occupations	90 s	
	C	tools	90 s	

^a^ADT and the associative combination task included three parallel forms (A, B and C).

### 2.3. The associative-dissociative task

The ADT is a novel experimental task, in which individuals retrieve words to serially presented word stimuli according to two specific conditions: FA and DA. Each trial started with a random word stimulus (e.g. ‘*coffee*’ (‘*káva*’ in original Slovak language)), presented either in green or red colour, designating which of the two respective conditions to follow: a green colour indicated FA trials, in which participants were to respond with the first related word (improper noun; e.g. ‘*cup’* (‘*šálka*’)) that came to their mind after seeing the stimulus, whereas a red colour indicated DA trials, in which they were required to retrieve an unrelated word (e.g. ‘*rope’* (‘*lano*’)) and instructed that delivering a semantically related word would count as an error. Each condition was shortly practiced (10 associative and 10 dissociative trials) before the main task. The main task consisted of two consecutive blocks, each including the same 40 stimulus words (the list of word stimuli for all three parallel forms is provided in appendix A, tables 5–7). The order of the stimuli was random for each block and participant. The retrieval conditions were pseudorandomized so that each block included exactly 20 associative (FA) and 20 dissociative (DA) trials, but the stimuli in the two blocks had the opposite retrieval conditions (i.e. if a stimulus word was presented in a green colour in one block, the same stimulus was repeated in a red colour in the other block), which was counterbalanced. The FA and DA trials were intermixed within the blocks to prevent participants from developing explicit response strategies, which happens when the order of experimental conditions is blocked and hence completely predictable. After the stimulus onset, participants typed a single response (word or short phrase, if needed) using a computer keyboard as quickly as possible. They were instructed to ignore grammatical or typing errors, and to minimize repeated responses within each block as much as possible. A blank screen was presented for 150 ms after each trial (response).

Each response was assessed for RT, i.e. the latency from stimulus onset to response initiation (pressing the first letter key of the intended word). IC was calculated as the ΔRT between the latency in DA and FA condition, separately for each word stimulus. Thus, there were three RT measures (FA, DA and IC) for every word stimuli and participant. Two independent raters screened the responses to individual stimuli within each condition (FA, DA) and form (A, B, C) for three types of errors: (i) rule violation error (i.e. responding with an unrelated word on the FA trial or with a related word on the DA trial, or responding with other than improper noun on both trials); (ii) repetition; and (iii) strategy error indicating the exploitation of deliberative strategy in responding (e.g. using the same initial letter or semantic category for cueing three or more successive responses). Final error designations were determined after mutual agreement between the two raters. On average (± s.d.) across the forms, 2.05 ± 2.65% of responses in the FA condition and 4.45 ± 5.30% in the DA condition were marked as rule violation errors. Repetitions occurred in roughly equal proportions between the retrieval conditions (FA = 2.98 ± 3.15%; DA = 3.10 ± 4.45%). Strategy errors were exclusive to DA trials (1.83 ± 3.96%). In total across the retrieval conditions, we removed 3.15% of responses as repetitions, 4.73% as errors (rule and strategy errors combined), and 3.72% as extreme latencies (RT > 3 s.d. above average) before processing the RT data. Note that individuals with greater than 30% error-rate across the responses were not included in the statistical analyses. Descriptive statistical analysis of the remaining raw data indicated the presence of outlying observations (values 1.5 interquartile range (IQR) below Q1 or above Q3) and thus, the RTs were winsorized (10% two-sided trimming by participant, form, and retrieval condition) and subsequently averaged, separately for each individual, to indicate their automatic (FA) and controlled (DA, IC) retrieval performance in each ADT form.

Three parallel forms (A, B and C) of the ADT task, including 40 unique words, matched in orthographic, lexical and psycholinguistic attributes (see table 2 for summary statistics), were constructed and administered to the participants across the testing blocks. ADT word stimuli were sampled from the Slovak National Corpus [45] (prim-9.0-public-sane: 1257 × 10^6^ words) to cover predominantly concrete improper nouns referring to concepts of everyday experience and with diverse corpus frequency (ranging from 15 × 10^3^ to 553 × 10^3^). Additional criteria for word selection included word length (number of letters) and polysemy (number of word's distinct meanings found in the lexicon). Next, as psycholinguistic attributes modulate the way words are processed (see [46–48]), the selected word stimuli were further assessed for word concreteness (abstract–concrete), imaginability (difficult–easy), contextual availability (low–high), emotional valence (negative–positive), and arousal (calm–arousing) using an ordinal rating scale (from 1 to 7; for more details on the scales, see [46]). Psycholinguistic ratings were obtained from an independent group of 53 individuals (31 females, mean age 23.8 ± 3.7 years) via an online rating form. Rating scales were presented in random order and words within individual scales were organized into 24 blocks (each containing five words) that were also randomly ordered for each participant. Participants were instructed to work alone in an undisturbed environment and to work with the first evoked meaning of the word. Participants were financially compensated after finishing the ratings. Ratings were averaged across the participants to estimate individual psycholinguistic properties for each stimulus word. These averaged ratings were subsequently subjected to PCA (see §2.5) and two composite scores were obtained: (i) *vividness* (high score = highly concrete, imaginable, and contextually available word) explaining 56% of the variance; (ii) *affective tone* (high score = words evoking positive affect and low arousal; low score = words evoking negative affect and high arousal) explaining 44% of the variance in psycholinguistic data (for more details, see the electronic supplementary material, figures S1 and S2).

**Table 2.** Summary statistics of lexical and psycholinguistic attributes for ADT stimuli by form. (Note: word length refers to the number of letters, and frequency to logarithm of word frequency in the national corpus database. Polysemy indicates the number of distinct meanings of a word found in the lexicon (the values represent medians (*M*) and IQRs). Vividness and affective tone are normalized *z*-scores derived from a principal component analysis. Vividness was indicated by concreteness, imageability, and context availability ratings, whereas affective tone was indicated by arousal and valence ratings. Omnibus tests were performed by one-way ANOVA and Kruskal-Wallis (polysemy) tests.)

attribute	form A		form B		form C		comparison
	*M*	s.d.	*M*	s.d.	*M*	s.d.	omnibus test
word length	4.78	1.03	4.82	1.11	4.92	1.07	*F* = 0.20, *p* = 0.81
word frequency	4.76	0.33	4.76	0.32	4.76	0.32	*F* = 0.01, *p* = 0.99
polysemy	2.00	1.25	2.00	1.25	2.00	1.00	*χ*^2^ = 0.05, *p* = 0.97
vividness	0.12	1.00	−0.16	1.14	0.04	0.99	*F* = 0.78, *p* = 0.46
affective tone	0.06	0.91	−0.07	1.05	0.01	1.17	*F* = 0.16, *p* = 0.85

### 2.4. Other cognitive measures

#### 2.4.1. Processing speed

Processing speed was assessed via three distinct tasks involving rapid information processing. In the *digit-symbol substitution task* [49], the participants were required to match eight distinct symbols to numbers according to a key located on the top of the computer screen. The symbols (stimuli) were presented randomly in the middle of the screen and participants responded using a keypress (numbers 1–8) while the RT was measured. The task included 16 practise and 64 test trials (equal number of trials for each symbol). The processing speed performance was calculated as winsorized average RT (10% two-sided trimming) from correct trials divided by the overall accuracy (*M*_Acc_ = 96.6 ± 3.4%). In the *letter matching task* [50], participants were simultaneously presented with a string including two target letters (e.g. ‘ER’) and a longer string of eight letters (e.g. ‘HDRMENZT’), displayed next to each other in the middle of the screen. In each trial, they were instructed to indicate whether at least one of the two target letters was present in the longer string using a keyboard. The task included 10 practise and 60 test trials presented serially and in random order. The performance was calculated as winsorized average RT (10% two-sided trimming) from correct trials including a match divided by the overall accuracy (*M*_Acc_ = 89.9 ± 8.1%). Finally, in the *choice response time task* (adapted from [51,52]), participants were presented with a series of white or red arrows (50% probability) pointing in four distinct directions (i.e. left, right, up, down; equal 25% probability). If the arrow stimulus was white, participants were instructed to press a keyboard arrow in the corresponding direction, while the red arrow indicated that participants were required to press a keyboard arrow in the opposite direction (e.g. to press the left keyboard arrow when seeing a rightward red stimulus). The task included 32 practise and 72 test trials. The processing speed was calculated as winsorized average RT (10% two-sided trimming) across all correct trials divided by the overall accuracy (*M*_Acc_ = 97.2 ± 2.6%).

#### 2.4.2. Working memory capacity

Individuals’ working memory capacity was assessed using three span tasks. In the *alpha span task* [53], participants were asked to remember a series of sequentially displayed random consonants and recall them in alphabetical order at the end of each trial. In the *operation span task* [54], participants remembered a series of sequentially presented random consonants but recalled them in the same order. However, after the presentation of each letter in the series, a simple equation was displayed on the screen (e.g. ‘37–14 = 23’), requiring the participants to indicate whether its solution is true or false using a keypress (the distractor task). Finally, in the *rotation span task* (e.g. [55]), participants were instructed to remember a series of random digits (0–9) and recall them in the same order at the end of each trial. After the presentation of each digit in the series, two rotated letters were displayed on the screen next to each other, requiring the participant to indicate whether the letters are rotated by the same or different magnitude (50% probability) using a keypress (the distractor task). The difference of rotation between the two letters ranged from ± 20 to 40°. In all three working memory tasks, the starting span was two items (digits or letters). After a successful recall, the span of the next trial was increased by one, up to the maximum of nine items. After each incorrect recall, the span for the next trial was decreased by one, to the minimum of two items. Participants were not allowed to rehearse the numbers out loud or by whispering during the tasks. Each task terminated after a total of five errors and the overall working memory capacity was calculated as the average span of the five incorrect trials minus 1.

#### 2.4.3. Associative combination task

Associative combination task was administered to assess associative combination ability (for a similar approach, see [28,36,56]). In each trial, a pair of words (e.g. ‘*city* + *river*’ (‘*mesto* + *rieka*’)) was presented in the middle of the screen, and participants were asked to deliver a word that relates to both stimulus words simultaneously (e.g. ‘*transport*’ (‘*doprava*’)) in a time limit of 15 s without repeating the same responses within each run (test form). The word pairs were constructed as all non-repeating combinations of 12 specific nouns, which resulted in a total of 66 stimulus pairs. Each parallel form (A, B, C) thus included a set of six practise and 22 unique test trials (word pairs). The response was counted as an error automatically if not delivered within the time limit of 15 s, or evaluated as erroneous by two independent raters if it did not comply with the rules of the task (i.e. responding with other than an improper noun, or with a word not related to both cue words). Final error designations were determined after mutual agreement between the two raters, following which 13.1% of responses across the parallel forms (A, B, C) were removed as errors and an additional 1.1% as having extreme latencies (RT > 3 s.d. above average). Then, the individual latencies were winsorized (10% two-sided trimming) by participant and task form and subsequently averaged for each participant in respective forms to indicate their associative combination performance.

#### 2.4.4. Semantic verbal fluency

To assess semantic verbal fluency, participants were asked to retrieve and type as many unique (i.e. not repeating) exemplars from specific categories as possible. The task included a short practice trial (*‘clothes‘*) and three test trials (categories: ‘*animals*‘, ‘*occupations*’ and ‘*tools*’) administered across the three testing blocks, respectively. Each verbal fluency task lasted 90 s. Two independent raters evaluated the responses from each category for inappropriate responses (out-of-category words, e.g. ‘*paw*’ (‘*laba*’) for animals category) and repetitions. In total, we registered 3.98% of responses as errors in the animals category, 1.57% in the occupations category, and 5.95% in the tools category. Response sequences containing more than 30% of errors were completely omitted from further analyses. Individual response sequences were then cleared of errors (2.33% across all categories) before counting the number of retrieved exemplars for respective categories to indicate individuals' verbal fluency performance.

### 2.5. Principal component analyses

Multiple measures of stimulus properties and cognitive capacities were used in the current study. To increase the robustness and reliability of the estimated variables of interest, we used PCA to extract the common variance (in the form of composite *z*-score) among the data related to (i) psycholinguistic and (ii) associative properties of the ADT stimuli, and (iii) to individuals’ performance across the cognitive tasks. These standardized *z*-scores were used to operationalize either the properties of the ADT stimuli (part 2 of the study) or individual differences in the corresponding cognitive capacities (part 3 of the study). Pearson correlation matrices served as an input for all PCAs. In each PCA, we determined the number of retained non-random components using the parallel analysis approach (95th percentile of *n* = 500 simulated eigenvalues). Component loadings were extracted using oblique rotation (oblimin) and in the case of multiple components, the *z*-scores were produced from a pattern matrix to capture only the unique variance of individual components.

## 3. Part 1: latency and reliability of the retrieval measures

Following the previous studies using similar kinds of retrieval tasks (e.g. [10,27]), we expected that the mean retrieval latency would be higher in the DA than FA retrieval condition. Furthermore, in contrast to the traditional verbal fluency tasks, only few studies have yet addressed the reliability of measures involving associative/dissociative retrieval [20,31,36,57] and a more systematic psychometric analysis was thus required. To this aim, the parallel ADT forms were examined for internal consistency (McDonald's *ω* [58]) and test–retest reliability, whereas the reliability estimates of 0.70 or greater were considered acceptable [59]. Moreover, using a random item sampling approach, we further investigated how these estimates change depending on the number of trials included in the ADT forms (up to 40 trials).

### 3.1. Results

#### 3.1.1. Retrieval latency

**Figure 1.**
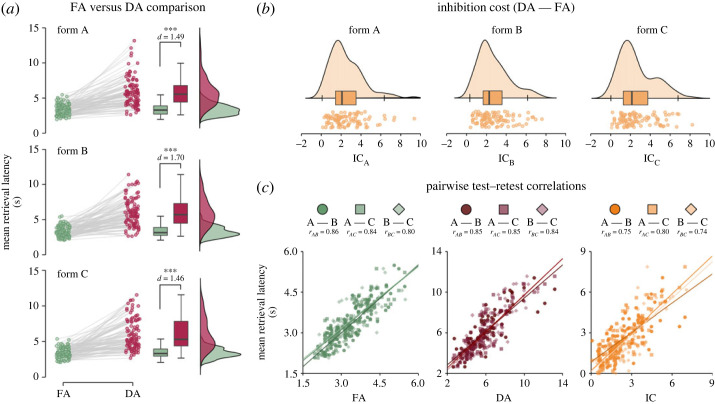
Performance and test-retest reliability in ADT across the parallel forms. Note: (*a*) indicates the differences between free-associative (FA; green colour) and dissociative (DA; red colour) retrieval latency, separately for each ADT form (A, B, C). Coloured point estimates in the plots represent individuals' mean retrieval latency in FA and DA, which are further summarized using box plots and density distributions of the corresponding colour (all differences were statistically significant at *p* < 0.001, whereas Cohen's *d* > 1.46); (*b*) shows individual data points of inhibition cost (IC, orange colour) and their distributions across the parallel forms (i.e. IC_A_, IC_B_, and IC_C_); and (*c*) visualizes pairwise (test-retest) correlations between the parallel forms for FA (green), DA (red), and IC (orange) using scatterplots. Data points for the pairwise correlations between the distinct forms are indicated in the scatterplots by specific marks (circle, square, diamond) and shades of the corresponding colour (all correlation pairs were significant at *p* < 0.001).

A repeated measures ANOVA for the ADT retrieval latency indicated a robust effect of retrieval condition, *F*_1.4,133.4_ = 389.781, *p* < 0.001, $\eta_{p}^{2}=0.802$, but negligible and statistically non-significant effect of task form, *F*_1.9,189.4_ = 1.151, *p* = 0.318, $\eta_{p}^{2}=0.012$, as well as their interaction, *F*_2.8,273.5_ = 2.232, *p* = 0.088, $\eta_{p}^{2}=0.023$ (for more details see figure 1 and table 3). *Post-hoc* paired-sample *t*-tests further confirmed that the latency differences between FA and DA retrieval conditions (i.e. the inhibition cost) were approximately similar across the forms, *t ≤* 1.822, *p*_Holm_ ≥ 0.215, *d* *≤* 0.123 (figure 1*b*). Similarly, mean FA retrieval latency, *t ≤* 2.342, *p*_Holm_ ≥ 0.063, *d* *≤* 0.142 and mean DA retrieval latency, *t ≤* 1.485, *p*_Holm_ ≥ 0.0423, *d* *≤* 0.081, were not significantly different across the parallel forms, indicating that the forms were equivalent with respect to the performance. Finally, as part of the exploratory analyses, we assessed whether the order of retrieval conditions in which the stimuli appeared during ADT (FA → DA versus DA → FA) had a significant effect on response latency in the respective tasks (i.e. whether retrieving first a related word to a stimulus impacted the subsequent retrieval of a semantically unrelated word or vice versa). The results indicated significantly slower retrieval in both conditions when the stimuli previously appeared in the alternative condition, and this effect was significantly larger for DA trials than FA trials (for more details, see the electronic supplementary material). Please note, however, that this effect should not present any systematic bias in the latency data as the order of the retrieval conditions in which word stimuli appeared was evenly counterbalanced within all ADT forms.

**Table 3.** Comparison between free-associative and dissociative retrieval latency in the parallel ADT forms. (Note*:* the descriptive measures represent response latencies (*M* ± s.d. in seconds) based on the individuals' data.)

form	retrieval condition		comparison				difference	
	FA	DA	*t*	d.f.	*p*	*d*	estimate	s.e.
A	3.35 ± 0.77	5.87 ± 2.06	−14.969	100	<0.001	−1.489	−2.511	0.168
B	3.30 ± 0.75	6.01 ± 1.99	−16.977	99	<0.001	−1.698	−2.720	0.160
C	3.41 ± 0.71	6.02 ± 2.16	−14.546	98	<0.001	−1.462	−2.610	0.179

#### 3.1.2. Reliability

The internal consistency of the three parallel forms was excellent for FA (*ω*_FA_ = 0.94), DA (*ω*_DA_ = 0.94), as well as IC (*ω*_IC_ = 0.91). The test-retest reliability between the form pairs, administered after approximately 30 to 60 min delay, was very high for FA (*r*_FA_ = 0.80–0.86) and DA (*r*_DA_ = 0.84–0.85). For IC, the strength of correlations between the parallel forms was slightly lower but still high (*r*_IC_ = 0.74–0.80; figure 1*c*).

**Figure 2.**
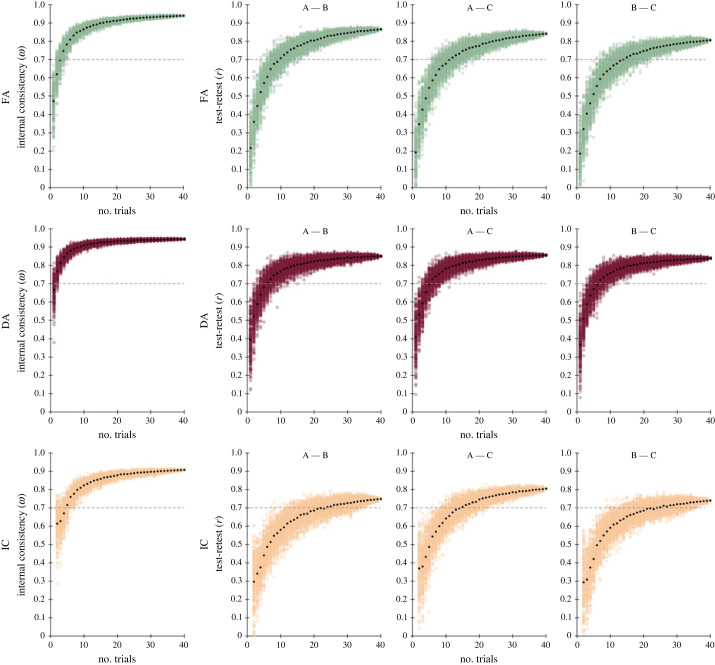
Predicted internal consistency and test-retest reliability of ADT measures. Note: the plots illustrate internal consistency and test-retest reliability estimates (*y*-axes) for free-associative retrieval (FA; top green plots), dissociative retrieval (DA; middle red plots), and inhibition cost (IC; bottom orange plots) as a function of the number of trials retained in the test (*x*-axis). The leftmost plots show internal consistency (McDonald's *ω*) across the ADT forms (A, B, C), whereas the other plots indicate test-retest correlations (Pearson *r*) between the form pairs (i.e. A–B, A–C, and B–C). In all plots, data points in colour represent reliability estimates (*ω* or *r*) of randomly sampled subsets of trials of a particular size (the maximum set size was 40 trials, which is the total number of trials included in the parallel forms): for every given set size (i.e. *x*-axis value), the corresponding number of trials was selected at random from each ADT form. Then, the average response latency of the selected subset of trials was calculated for each participant and form to indicate the retrieval performance, which was subsequently assessed for internal consistency and test-retest correlation. This trial-sampling procedure was repeated 200 times to obtain a distribution of reliability estimates (note that in each repetition a different subset of random trials could be selected). Finally, the average reliability of those 200 individual reliability estimates was calculated (indicated by black data points) for every set size. As demonstrated in all plots, the average reliability increases with the number of trials retained in ADT forms. More importantly, these plots provide valuable information regarding how many trials are necessary to achieve a desired level of reliability (the grey dashed line indicates the threshold for acceptable reliability, i.e. *ω* and *r* of 0.7 or above).

Furthermore, we used a random trial sampling procedure to evaluate how the number of trials (i.e. RT values) included in ADT affects the reliability of its measures. The procedure included several runs with a gradually increasing number of trials being analysed, starting with only *n* = 1 trial up to the maximum of *n* = 40 trials (i.e. the total number of trials included in each ADT form). In each run, only a subset of exactly *n* trials was selected at random from each parallel form and retained. This subset of trials was then used to calculate the participants' retrieval performance in each form (i.e. average retrieval latency) and the reliability estimates (i.e. McDonald's *ω* and test-retest Pearson correlations). Importantly, this random trial sampling was repeated 200 times within each run to obtain a distribution of reliability estimates (i.e. in each of the 200 repetitions the corresponding subset of *n* trials was selected at random for each parallel form, and therefore these individual reliability estimates vary). Finally, the average reliability was calculated from the 200 reliability estimates in each run. Consequently, using this procedure we generated 200 reliability estimates (and their average) in each of the 40 runs, separately for each reliability (*ω* or r) and retrieval measure (FA, DA, and IC). These data are visually summarized in figure 2.

In the last step, we computed a Cronbach-Mesbah curve (CMC), which represents a simple yet effective method for evaluating the unidimensionality of a measurement tool and selecting the best performing items/trials (see [60]). The CMC indicated that all three ADT measures are unidimensional. Moreover, this analysis also indicated that the clinical level of reliability (i.e. *r* > 0.90; [61]) can be achieved using only up to 25 items, provided that psychometrically best-performing items are preferentially selected (see the electronic supplementary material, figure S2 for more details).

### 3.2. Interim summary

The first part of the study evaluated the retrieval latency and reliability of the ADT measures. Replicating previous research [10,40], we demonstrated that retrieving unrelated concepts (DA condition) requires substantially more time compared to free-associative retrieval (FA condition), which has been interpreted in terms of increased demands on inhibitory control of retrieval [27,29,34]. Importantly, the retrieval latencies in FA and DA conditions, as well as their difference reflecting the IC, were approximately identical and strongly correlated across the parallel forms (figure 1). These findings thus suggest that ADT measures are highly reliable and putatively reflect trait-like cognitive abilities (capacities). We also showed that all three retrieval measures in ADT demonstrate very good temporal stability for up to 60 min, rendering ADT suitable for research designs adopting repeated measurements, at least when administered within a single session.

## 4. Part 2: stimulus-level determinants of the retrieval performance

Our next goal was to provide researchers with a standard set of word stimuli (items/trials) and evaluate which of their properties affect the retrieval performance. Critically, certain lexical and psycholinguistic properties of words, such as length or familiarity, may impact the way they are processed and predict the involvement of specific neurocognitive resources during memory retrieval [62,63]. Several studies have demonstrated that the processing of concrete words benefits from higher imageability and context availability, since they have more distinctive sensorimotor features compared to abstract words [46,64,65]. Furthermore, compared to words with single/dominant meaning, the processing of polysemous words exerts greater demands on interference control and response selection [66], and emotional valence and arousal that individuals tend to associate with words also appear to modulate word processing and recognition [67,68]. Drawing upon such evidence, these psycholinguistic dimensions were assessed and expressed in vividness and affective tone scores.

The processing of words is putatively also influenced by their associative properties, which can be described in terms of associative hierarchies (i.e. a stimulus-evoked set of typical associative responses ordered by their relative strength) and quantified using word association norms [69]. Previous studies have suggested that retrieval fluency and originality of responses are predicted by steepness (i.e. the relative decrement in associative strength) and topology (mutual relations) of associative hierarchies [17,31,70,71]. To assess these stimulus dimensions, study participants were asked to provide four associations (improper nouns) to each ADT stimulus via an online form at the end of the main procedure and instructed to complete the form within two days after attending the session. We reminded the participants to avoid association chaining (i.e. associating to a previously entered association on a given stimulus) as previous entries remained visible during the task. The list of stimulus words was randomly reshuffled for each participant. Collected associations were processed by removal of inappropriate words (i.e. other than improper nouns), removal of words with frequency of less than 2, and by transformation of synonyms to common terms (to minimize the dilution of word occurrences). The resulting association data included on average (± s.d.) 355 ± 12 word association responses to each ADT stimulus.

Frequency counts of the provided associations were subsequently used to estimate the following variables for each ADT stimulus word: (i) proportional occurrence of the strongest association calculated as the ratio between the frequency of the most frequent association and summed frequencies of all associations; (ii) frequency decrease slope (*β*) calculated by regressing the order of the 10 most frequent associations (1 to 10, from most to least frequent) onto their respective frequencies; and (iii) frequency intercept estimating the average frequency of the 10 most frequent associations. The purpose of these variables was to differentiate stimulus words with a very steep associative hierarchy (evoking a highly typical and predictable set of few dominant associations) from those with flatter associative hierarchy (where the produced associations are less typical and predictable, i.e. no specific associations stand significantly above the others).

In the next step, we used the collected associations to model a weighted undirected network for each ADT stimulus word. In these networks, nodes represented unique associations provided to a given stimulus word whereas links indicated associative/semantic connections between these associations. The weights were quantified using LogDice similarity scores, which consider the raw collocations of two associations (counted each time two associations co-occurred within the sequence of four associations to a given stimulus) and their respective occurrences within the association norms (see [72]). Note that LogDice was selected to quantify associative weights in networks as this metric is predictive of response latencies in various semantic tasks [31,32,73]. Then, four variables describing the associative topology of the networks were calculated: (i) *modularity* (Louvain algorithm [74]) quantifying the partitioning of word associations into distinct semantic clusters; (ii) *average shortest path length* [75] expressing the weighted distances among all associations in the network; (iii) *global clustering coefficient* indicating the tendency of associations linked to any other association in the network to be also connected; and (iv) *global network efficiency* [76,77], indicating the degree to which a network affords efficient propagation of information among its nodes. Variables were estimated by functions implemented in ‘NetworkToolbox’ and ‘brainGraph’ R packages [78,79]. This allowed us to further differentiate stimulus words based on the efficacy of their associative topology to either facilitate (densely connected associations with short distances among them) or hinder (sparsely connected associations with long distances among them) the spread of activation across their associative neighbourhood.

Similarly, the dimensionality of these data was reduced using PCA to extract two composite scores describing associative properties of ADT stimulus words: (i) *associative typicality* (high score = stimulus evokes a small set of highly typical/frequent associations followed by a steep decrease in the frequency of subsequent associations; low score = stimulus evokes less typical associations of flatter frequency distribution); and (ii) *associative topology* (high score = stimulus evokes a highly interconnected set of associated responses; low score = stimulus evokes sparsely connected responses that are partitioned into distinct semantic clusters). The components explained an equal portion of the variance (i.e. 50%) in the data (for more details see the electronic supplementary material, figure S1 and table S2; also figure S3 visually demonstrates high and low associative typicality and topology scores on four example stimulus words).

Based on previous research [27,70], we hypothesized that by evoking a few highly dominant associates, stimuli with high associative typicality would benefit free-associative retrieval but simultaneously increase demands on inhibitory capacities. Similarly, we expected that stimuli evoking a set of mutually related responses (i.e. high associative topology score) would facilitate the search for candidate word associations and hence aid the free-associative retrieval. On the other hand, however, strong mutual connections among the typical responses may conduct semantic activation among task-irrelevant competitors and so impair the dissociative retrieval. Following this rationale, we expected word stimuli with high associative typicality and topology scores to facilitate free-associative retrieval but impair inhibition. The effects of vividness and affective tone scores on retrieval performance were investigated in an exploratory manner.

### 4.1. Results

**Figure 3.**
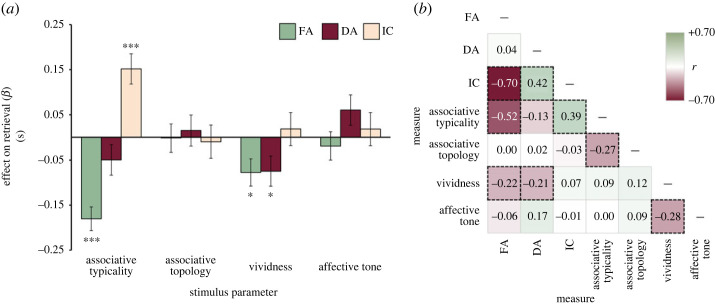
Stimulus-level effects on semantic memory retrieval. Note: (*a*) estimated effects of stimulus properties on the retrieval performance in ADT. The bars depict changes in retrieval latency (in seconds) when the corresponding stimulus parameter (*z*-scores) changes by one unit (negative values indicate better retrieval performance). These estimates were obtained by including the respective stimulus-level factors in LMEMs to predict the retrieval performance (*p < 0.05, ***p < 0.001); (*b*) correlations among retrieval latencies and stimulus-level measures aggregated across all participants. To conduct this analysis, the individual response times from all participants were averaged to estimate the mean free-associative latency (FA), dissociative retrieval latency (DA), and inhibition cost (IC) for each word stimulus in ADT. These stimulus-level latency estimates were then correlated with the psycholinguistic and associative parameters (i.e. associative typicality, associative topology, affective tone, and vividness) across all 120 stimuli. Dashed black squares mark statistically significant correlations.

Effects of psycholinguistic and associative variables on FA, DA and IC were tested by separate linear-mixed effect models (LMEM) which included random intercepts for participants and stimulus words. Stimulus associative typicality had a substantial and significant impact on free–associative latency, indicating that participants responded faster to stimuli with high than low associative typicality score, *β* = −0.180 (s.e. = 0.026), *t*_116_ = −6.844, *p* < 0.001. The effect of associative typicality on dissociate latency was not significant, *β* = −0.049 (s.e. = 0.034), *t*_114_ = −1.463, *p* = 0.146. However, higher typicality score predicted significantly larger inhibition cost, *β* = 0.152 (s.e. = 0.034), *t*_114_ = 4.518, *p* < 0.001 (figure 3*a*). The associative topology parameter had no significant effect on any retrieval measure (*p* > 0.656). Items' vividness score predicted both faster associative latencies, *β* = −0.077 (s.e. = 0.030), *t*_118_ = −2.554, *p* = 0.012, and dissociative latencies, *β* = −0.075 (s.e. = 0.033), *t*_118_ = −2.237, *p* = 0.027, in comparable size, but had no effect on the inhibition cost measure, *β* = 0.019 (s.e. = 0.036), *t*_117_ = 0.511, *p* = 0.611. Items’ affective tone score had no significant impact on any retrieval measure (*p* > 0.076).

Furthermore, we aggregated the data for each stimulus and assessed the correlation between the stimulus properties and retrieval latency. The correlations on stimulus-aggregated data confirmed the strong and negative effect of associative typicality on free–associative latency (*r* = −0.517, *p* < 0.001), non-significant effect on dissociate latency (*r* = −0.133, *p* = 0.146), and moderate positive relationship with inhibition cost (*r* = 0.393, *p* < 0.001), whereas associative topology showed no association with latency measures (*r* < 0.027, *p* > 0.767; for more details, see figure 3*b*).

### 4.2. Interim summary

The second part of the study represents an in-depth stimulus-level analysis investigating the effects of psycholinguistic and associative stimulus properties on retrieval fluency. We found that increasing associative typicality was coupled with faster free–associative retrieval but prolonged inhibitory processing. These effects validate the ADT measures by showing that retrieving related concepts is easier in trials involving stimuli that have few but very strong associates (i.e. high typicality score). On the other hand, however, triggering of such prepotent associates may impose additional load on inhibitory capacity, which accounts for the increased latency of their suppression [27,30]. Notably, associative typicality had no significant effect on the dissociative retrieval as such, which is likely because generating dissociates shares some processes with the free–associative retrieval (e.g. reading, processing of word meaning, activation of lexical-semantic memory representations, response initiation and monitoring; also see [40,80]) but also includes the inhibitory processes. Notably, associative topology and affective tone of word stimuli did not reliably predict the retrieval performance.

## 5. Part 3: convergent and discriminant validity of associative-dissociative task measures

The last goal of the present study was to evaluate the construct validity of the ADT retrieval measures. To this aim, we assessed four additional cognitive capacities (constructs), two of which involve semantic processing and long-term memory retrieval (i.e. verbal fluency and associative combination tasks [36,56,81]), whereas the other two represent domain-general cognitive capacities that do not engage such processes (i.e. processing speed and working memory tasks [82]). Individuals' performance data (*n* = 102) on three corresponding tasks or parallel forms were parsed into separate PCAs for each cognitive construct (i.e. there were three indicators per construct). Indicators of respective constructs yielded reliable and consistent loadings on a single component explaining 89% of the variance among the free-associative, 90% of the variance among the dissociative, 84% of the variance among the inhibition-cost, 67% of the variance among the verbal fluency, 79% of the variance among the associative combination, 65% of the variance among the processing speed, and 68% of the variance among the working memory performance (for more details, see the electronic supplementary material, figure S1 and table S2).

Following these theoretical differences, we expected moderate to strong correlations of ADT composite scores (FA, DA, IC) with semantic verbal fluency and associative combination composite scores, which would support the convergent validity of the ADT measures, but null or only weak correlations with processing speed and working memory composite scores, which would support their discriminant validity. Furthermore, assuming that the retrieval in semantic verbal fluency and associative combination task may partially rely on inhibitory processes [40,80], we expected that smaller IC would predict better performance in these tasks. Finally, as ADT measures predict greater reliance on semantic and associative capacities for associative than dissociative retrieval (e.g. [40]), we expected the FA composite score to be more strongly coupled with verbal fluency and associative combination than the DA composite score. To test these predictions, we evaluated statistical differences between the corresponding correlation coefficients (Δ*r*) using the Fisher *Z* transformation method [83].

### 5.1. Results

The relationships among these scores were examined using Pearson correlations (table 4). This analysis indicated no statistically significant correlations between the retrieval measures in ADT and working memory capacity (|*r*| < 0.125, *p* ≥ 0.209), whereas processing speed significantly predicted the free-associative retrieval (*r* = 0.265, *p* = 0.007) but not the dissociative retrieval (*r* = 0.114, *p* = 0.264) or the inhibition cost (*r* = 0.015, *p* = 0.882). Furthermore, as expected, higher verbal fluency performance correlated with shorter latencies in the free-associative retrieval (*r* = −0.696, *p* < 0.001), the dissociative latency (*r* = −0.559, *p* < 0.001), and smaller inhibition cost (*r* = −0.376, *p* < 0.001) in ADT. Finally, the associative combination ability positively predicted the free-associative performance (*r* = 0.713, *p* < 0.001), the dissociative performance (*r* = 0.479, *p* < 0.001), as well as the inhibition cost (*r* = 0.322, *p* < 0.001). Importantly, we found that, compared to dissociative performance, free-associative performance was significantly more coupled with verbal fluency (Δ*r* = −0.137 [95% confidence interval (CI): −0.427; −0.030], *Z* = −2.252, *p* = 0.024) as well as associative combination performance (Δ*r* = 0.221 [95% CI: 0.159; 0.551], *Z* = 3.546, *p* < 0.001).

**Table 4.** Correlations between component scores of ADT measures and cognitive capacities. (Note: Pearson correlations; **p < 0.01, ***p < 0.001. Component scores for individual constructs reflect capacity for working memory, response time for processing speed and associative combination, and number of retrieved words for semantic verbal fluency.)

construct	free-associative retrieval	dissociative retrieval	inhibition cost
working memory	−0.125	0.038	0.118
processing speed	0.265**	0.114	0.015
semantic verbal fluency	−0.696***	−0.559***	−0.376***
associative combination	0.713***	0.497***	0.322**

### 5.2. Interim summary

In this part of the study, we further examined the construct validity for all three ADT measures. As hypothesized, all measures involving semantic memory retrieval were moderately related to each other, supporting their convergent validity. This correlation pattern was expected since the retrieval tasks involve a set of shared domain-specific (i.e. verbal/lexical-semantic) as well as domain-general abilities. Yet, associative combination and semantic verbal fluency performance were somewhat more strongly coupled with the free–associative retrieval than with the dissociative retrieval, supporting the idea of distinct reliance of FA and DA retrieval on richness and connectedness of semantic knowledge, which drive the performance in verbal fluency and associative combination tasks (for similar findings, see [36,40]). Furthermore, the ADT measures were not reliably related to individuals’ processing speed and working memory capacity, which support their discriminant validity. In fact, the only exception was the positive but weak correlation between the free–associative retrieval and processing speed, showing that rapid information processing and psychomotor tempo may be more apt for less complex (i.e. automatic) rather than executively demanding (i.e. controlled) retrieval [51]. Next, although the retrieval from semantic memory was previously found to benefit from adaptive maintenance and executive updating of retrieval cues in working memory (see [73,84]), these effects may be specific to retrieval performance in the classical category fluency tasks that rely on an optimal exhaustion of semantic clusters (i.e. clustering) and flexible transitions across distinct clusters encoded in semantic memory (i.e. switching [85]). Since responding with a single word in ADT precludes such temporal-semantic dynamics, the contribution of working memory capacity to free-associative and dissociative retrieval is probably less relevant. However, keeping the goal of memory search in focus and shielding it from distractions may still play an important role, particularly for dissociative retrieval (see [51]). Therefore, further research implementing a fine-grained manipulation of specific working memory components (i.e. central executive and phonological loop) and functions (e.g. maintenance, updating and information processing) is required to evaluate the causal involvement of this cognitive system in the retrieval processes probed by ADT.

## 6. General discussion

Owing to their central role in human adaptive behaviour, cognition and language, the processes involved in semantic memory retrieval have earned extensive research interest during the last decades [2,86,87]. Spanning behavioural, neuroimaging or computational studies, it has been determined that memory retrieval provides robust, but also highly flexible inferences about the environment by fast-acting associative (automatic) and slower, dissociative (controlled) processes acting upon the stored memory representations [11,13,88]. Extensive effort has been developed to capture these processes, yet attempts to establish psychometrically sound methods met with difficulties, often stemming from limited ability to control varying task-demands or other stimulus-inherent confounds [2,40,89]. With this in mind, we introduced and evaluated a novel ADT*,* developed to assess free-associative and controlled (inhibitory) semantic retrieval processes with three major goals to (i) evaluate internal consistency and reliability of the proposed measures, (ii) assess items' psycholinguistic attributes and estimate their effects on the proposed measures, and (iii) demonstrate convergent and divergent validity of the proposed measures against other semantic and domain-general executive tasks.

First, we established and replicated the essential distinction between the associative and dissociative retrieval by showing that producing semantically unrelated response to verbal stimulus requires a significantly greater amount of time compared to producing related response, putatively reflecting increased demands on inhibitory control over task-irrelevant competitors [37,39]. Notably, the pattern of this distinction was roughly identical across the three parallel forms of the ADT (figure 1*a*,*b*). Importantly, both associative and dissociative retrieval latencies, and the derived inhibition-cost estimate demonstrated high internal consistency and test-retest reliability over the separate blocks and task forms. These findings demonstrate that ADT provides consistent information about the underlying memory retrieval capacities and suggest that the demands on automatic versus controlled semantic processing remain invariant across the ADT forms. Intriguingly, further calculations indicated that sufficient levels of reliability can be achieved by including only a fraction of the items (i.e. approximately 10–15 of the items in the case of associative and dissociative latencies, and by approximately 20–25 of the items in the case of inhibition cost; see figure 2). This parsimony allows flexible adjustments of the ADT to satisfy needs of experimental designs that may require several repeated measurements while not sacrificing much measurement precision and control over confounds owing to unequal task-demands. Notably, however, if a research goal does not require a balanced allocation of multiple items across several experimental conditions, one can select only the best performing items from the provided set and so surpass the strict standards for clinical assessment (see the electronic supplementary material, figure S2).

Furthermore, our stimulus-level analyses indicated that items’ vividness had a facilitative effect on both associative and dissociative retrieval, suggesting that high concreteness, imageability, and contextual availability of the verbal stimuli aids the identification of relevant semantic features, which may help to navigate the semantic search towards (associate) or away from (dissociate) close semantic neighbours [64,65]. It should be noted, however, that the distribution of vividness scores showed a celling effect, possibly attenuating the effect on the performance as a consequence. Furthermore, although the retrieval latency was not predicted by the affective tone, frequency, and polysemy, these properties may nevertheless modulate neuronal activity underlying word recognition and selection, especially in individuals with impaired semantic processing [48,66–68,90]. Crucially, as expected, items' associative typicality substantially enhanced the free–associative retrieval and prolonged inhibition costs. These findings corroborate the previous theoretical proposals (e.g. [36,91]), according to which steep associative hierarchies constrain the semantic activation within a narrow scope of prepotent/close associates, hence accelerating the associative retrieval but simultaneously increasing the inhibitory demands when attempting to disentangle from such habitual response candidates. Notably, items’ associative typicality did not modulate the dissociative performance. This is not that surprising since the dissociative retrieval not only involves the unique demands on inhibition, which are negatively affected by the associative typicality parameter (figure 3), but also shares a number of processes with the associative retrieval, which is positively predicted by the typicality parameter. Finally, we found no evidence for the effect of items' associative topology on the ADT measures. This contradicts the view that inhibition should be negatively impacted by high network connectivity of the verbal stimulus [27,70]. Specifically, by affording wide-spread semantic activation across the associative network, highly connected networks may prime a larger set of concepts, hence inducing higher interference and demands on inhibitory control. This inconsistency may originate from the fact that the few previous studies estimated semantic network topology on the level of individuals [27,77], while in the present study, we worked with the item-level sample-aggregated estimates. Further research is required to address the relationship between the efficiency of information spreading over semantic networks and retrieval abilities.

Finally, a critical test of the convergent and divergent validity revealed that the ADT measures coupled with the performance from the other long-term declarative memory tasks involving semantic processing (verbal fluency and associative combination) but not (or only marginally, see table 4) with the performance in domain-general executive tasks (processing speed and working memory). These results lay further support for the claim that the ADT performance measures are driven primarily by the long-term semantic memory capacities. Furthermore, the tasks involving semantic processing better predicted the FA than the DA performance, which closely mimics the findings from a recent latent modelling study [40]. Together, these results indicate that the free–associative retrieval is more substantially driven by the representational aspects of semantic cognition (i.e. the habitual taxonomic and thematic relations), whereas the dissociative retrieval more specifically reflects the controlled aspects of the lexical-semantic retrieval (also see [92,93]). Notably, since the dissociative production was not considerably saturated by domain-general executive capacities, it is possible to speculate that the dissociative (inhibitory) retrieval reflects domain-specific rather than domain-general control processes or task difficulty [82,88,94]. This proposal is also supported by previous studies showing that the ability to suppress habitual but task-irrelevant retrieval candidates engage lateral prefrontal and posterior temporal regions implicated in the semantic control [10,29,37,39,95]. Taken together, apart from displaying exceptional measurement reliability and sensitivity to psycholinguistic/associative aspects of word stimuli, our findings also confirm the construct validity of the ADT measures for assessing the automatic (free–associative) and controlled (inhibitory) lexical-semantic retrieval abilities.

### 6.1. Related cognitive tasks

By its generative nature, the ADT shares common characteristics with other retrieval paradigms, most prominently with verbal fluency tasks (i.e. both target active search and open-ended retrieval of lexical-semantic knowledge). While traditional verbal fluency tasks provide a fast and easy way to gauge verbal abilities and memory, their capability to rigorously track separate involvement of automatic versus controlled retrieval is poor [81,96,97]. Usually, category- and letter-fluency tasks are separately used to test functionality of automatic and controlled components of lexical-semantic retrieval. Yet, research also continues to point out that differences between category- and letter-fluency are parametric rather than absolute, with both containing traces of executive and associative involvement [96,98,99]. This excludes the possibility of using contrasts between category and letter fluency performance as indicators of costs associated with retrieval control demands. Beyond that, stimuli in fluency tasks (being it different categories or letters) suffer from stimulus-inherent confounds (e.g. number, frequency or taxonomic and thematic clarity of candidate words) which influence retrieval demands [32,89,100]. Another line of evidence suggests that common fluency measures of automatic (clustering) and controlled (switching) retrieval both capture executive processing to some degree, and the picture gets even blurrier when considering time spent performing the task [28,32,85,101]. All these shortcomings challenge the psychometric appropriateness of the verbal fluency tasks in providing specific information about automatic and controlled semantic processing. Consequently, using fluency tasks in repeated-measures designs carries uncertainty whether distinct categories (e.g. *animals* versus *tools*) exert comparable demands on the automatic versus controlled processes, or more generally, whether their idiosyncratic features afford for the involvement of distinct cognitive resources. The ADT overcomes these possible drawbacks by demonstrating high internal consistency and test-retest stability of the estimates related to automatic and controlled retrieval while using distinct, yet balanced set of stimuli. Moreover, since categories include a relatively narrow and finite set of possible retrieval candidates (e.g. ‘*tools*’ or ‘*fabrics*’), the retrieval difficulty gradually increases as the category exemplars become exhausted and participants can produce only a limited number of responses (for a recent discussion, see [32,40]). On the other hand, the number of response candidates available in the ADT tasks is in principle unlimited, which mitigates the problem of changing retrieval demands and other methodological confounds. Notably, akin to verbal fluency tasks, evaluation of lexical and semantic features of the responses in ADT (e.g. frequency, semantic or phonological similarity) may provide an option, beyond primary latency measures, to further disentangle possible sources of semantic deficits symptomatic to various neuropsychiatric conditions (for a similar approach, see [102] or [27]). Finally, compared to ADT, verbal fluency tasks tend to be more strongly related to the domain-general executive processes (e.g. working memory, monitoring or interference control) and thus may be indicative of an interaction between semantic memory retrieval and such processes. Nevertheless, semantic verbal fluency is a well-established measure in cognitive science and neuropsychology that provides very useful information on semantic memory retrieval, especially with respect to category-specific representations and deficits.

From yet another perspective, the ADT shares several theoretical assumptions and structural features with the well-established cognitive conflict paradigms, such as the Stroop task or the Simon task, to mention a few. The conflict tasks typically involve two conditions, congruent and incongruent, in which the processing of task-relevant and task-irrelevant stimulus features activate distinct responses that compete for execution, and participants must suppress the irrelevant contents or features to deliver a correct response (see [103]). Similarly, the ADT includes two conflicting conditions evoking response candidates that are either task-relevant/congruent (i.e. the FA condition) or task-irrelevant/incongruent (i.e. the DA condition). By this virtue, the inhibition cost (IC) from ADT can be conceptualized as a measure indicating the inefficiency of conflict resolution in the lexical-semantic domain. Although our behavioural data are compatible with this account, further electrophysiological and neuroimaging research is needed to investigate whether the neural mechanism subserving the detection and resolution of cognitive conflict (e.g. theta-band oscillations, see [104]) is differentially engaged during the respective retrieval tasks in ADT, or whether this engagement can be reliably predicted from inhibition cost specifically. Furthermore, in contrast to the standard conflict tasks that involve forced-choice responses with pre-established alternatives, our paradigm implements an open-ended response format that affords for the assessment of other qualitative dimensions of responses (e.g. typicality and semantic distances). Thanks to that, unique and informative data, beyond those contained in the IC measure alone (i.e. ΔRT), can be derived from the constituent FA and DA conditions and modelled using advanced computational approaches that have been recently successfully applied to data from various conflict tasks (e.g. [105,106]).

The ADT may also find its application in the domain of creative cognition. Various divergent-thinking tasks assume both spontaneous processes, mediating swift foraging through semantic network, and controlled processes, preventing fixations on prototypical meaning of a concept (but see [107] for a comprehensive review). For instance, Benedek and colleagues [36] found that associative abilities, especially associative combination and dissociation, explain approximately half of the variance of divergent thinking ability, suggesting their substantial contribution to creative ideation (also see [9,20,38]). Similarly, previous research has shown that individuals responding with less typical and more distant associations in the FA trials are more successful in solving remote associates test problems [31], suggesting that the assessment of semantic distances using ADT responses may provide means to approximate breadth of semantic search or dispersion of semantic activation, which is highly relevant to high-order cognitive phenomena such as creativity [38,108,109].

Taken together, in this methodological discussion, we identified several noteworthy differences between the ADT and previously established retrieval measures (e.g. verbal fluency or divergent thinking), emphasizing the dominant features and limitations that researchers should consider in the light of their specific research intents.

### 6.2. Limitations and future directions

Several limitations of the present study should be considered to pave future research. Although this study provided experimental and correlational evidence supporting the reliability and validity of the main ADT measures, the mechanisms underlying the associative and inhibitory functions during semantic memory retrieval has not yet been precisely described (but see [9,10,27,52]). More evidence is required to explore the proposed complementary processes pertaining to (automatic) semantic activation and (controlled) inhibition, particularly in the face of the prevailing ‘map and vehicle’ problem, which concerns what portion of performance variation can be attributed to differences in memory representations versus differences in processes that operate on them [110]. Moreover, further studies are necessary to address whether other controlled processes beyond inhibition (or interference control) support the dissociative retrieval and hence affect the inhibition cost measure. Contrary to the free-associative retrieval where individuals are encouraged to respond with the first concept that is evoked by the stimulus, the dissociative condition was constructed to require the opposite–i.e. to suppress the activation of such prepotent and typical retrieval candidates in memory, which, by definition, exerts demands predominantly on the inhibitory control mechanisms (see [39]). In line with this process-based account, individuals often report that typical word associates intrude into their minds while attempting to retrieve unrelated responses. Although the precise rate of intrusions awaits a formal investigation, our preliminary estimates suggest that such conceptual intrusions are present in most dissociative trials (for a similar account, see [111]). Notably, the assessment of intrusions in ADT would also allow for a more fine-grained analysis of the nature of inhibitory processes employed in the dissociative task since either a proactive inhibition (engaged to prevent the automatic conceptual intrusions), a retroactive inhibition (employed in a post hoc manner to suppress the conceptual contents that have already intruded the mind), or a mixture of both may be involved. Alternatively, other means of cognitive control may support fluid retrieval of weakly related concepts in the dissociative task. For instance, it can be speculated that an executive switching mechanism can be engaged to aid flexible transitions between semantic categories, hence mitigating the stimulus-evoked conceptual fixation [51] or facilitating the lexical access to remote conceptual representations [11]. Moreover, domain-general mechanisms responsible for conflict monitoring and implementation of cognitive control might be also necessary as the demands on semantic search become high (see [32,51]).

Finally, although the applicability of ADT measures in clinical settings is beyond the scope of the present study, several recommendations and suggestions can be considered based on the provided arguments and data. ADT seems generally well-suited for administration in various patient groups since the constituent retrieval tasks are easy to comprehend and relatively short (i.e. a test version tailored to include 30 free-associative and 30 dissociative trials can be completed in approximately 5–8 min and still offers excellent measurement precision). However, oral responding (spoken word production) in the retrieval tasks would be more appropriate in clinical assessment, especially in older individuals with insufficient typing skills or in patients with motor impairment. Even though an explicit comparison of oral versus written test form of ADT awaits further investigation and some differences may be expected, altering the response format should not challenge the validity or clinical use of the retrieval measures. For instance, the free-associative retrieval employs an unconstrained memory search that follows the habitual structure of semantic representation (i.e. overlearned conceptual patterns). Thanks to that, we hypothesise that this task may offer a sensitive way to study disturbances of conceptual stores (i.e. aberrant organization of conceptual elements in memory), which are frequently observed in mental health disorders. Moreover, the free-associative retrieval is also suitable for the assessment of spontaneous thought dynamics, which are altered in a wide range of clinical conditions (for a review, see [112]). On the other hand, we hypothesise that the dissociative retrieval task offers an intriguing way to assess the executive regulation of memory search, especially the neurocognitive mechanisms subserving the inhibition (i.e. suppression of prepotent but inappropriate responses) and interference control (i.e. resolving conceptual and response interference from competing concepts) which are often compromised in neuropsychiatric patients [113]. Relatedly, assessing the rate of intrusions encountered by individuals while delivering unrelated responses may represent a theoretically sound measure with the potential to provide deeper insights into psychopathological mechanisms underlying deficient thought control. Taken together, the proposed retrieval measures are highly relevant for many neuropsychiatric conditions, yet further research is needed to confirm the expected use of ADT in clinical research and application.

## 7. Conclusion

We have demonstrated that ADT incorporates promising measures that bear strong potential to find utility across a wide scope of cognitive domains, including research on memory, language, thinking, and creativity. Critically, by enabling the differentiation between automatic (free–associative) and controlled (inhibitory) processes, ADT may shed new light on the mechanisms supporting semantic search and retrieval control. Furthermore, we have provided a set of stimuli, described for psycholinguistic and associative features, as well as behavioural outcomes, to facilitate the customization of the task and item selection. In addition, parallel, balanced forms were prepared and are ready for immediate use. Neuroimaging and neurostimulation studies are needed to further elucidate the neurobiological basis of the ADT measures, whereas research in neurological and psychiatric patients may pave their application in clinical settings.

## Data Availability

The datasets generated during and/or analysed during the current study are available from the corresponding author upon reasonable request. The data, additional materials and codes are also openly available in the OSF repository (https://osf.io/z98my/) [114]. We report how we determined our sample size, all data exclusions, all manipulations, and all measures in the study. The study was not preregistered. Supplementary material is available online [115].

## Appendix A

**Table 5.** List of ADT word stimuli (form A). (Note: frequency represents logarithm of word frequency in the national corpus database and polysemy indicates the number of distinct meanings of the word found in the lexicon. Vividness, affective tone (high = positive and calm; low = negative and arousing), associative typicality, and associative topology (high = efficient; low = inefficient) are expressed in composite *z*-scores obtained from principal component analyses.).

stimulus word (SK)	frequency	polysemy	vividness	affective tone	associative typicality	associative topology
coffee (káva)	4.84	1	1.00	0.65	−2.16	1.76
blood (krv)	5.19	2	1.27	−0.43	−1.80	−0.43
tea (čaj)	4.74	2	0.00	2.06	−1.68	−0.29
factory/race (závod)	4.91	2	−3.36	−0.11	−1.35	−0.41
cancer (rak)	4.50	3	0.22	−0.48	−0.89	2.08
eagle (orol)	4.20	2	0.80	−0.42	−0.87	1.64
cellphone (mobil)	4.74	1	1.55	−0.46	−0.85	0.16
stand/stall (stánok)	4.64	2	−0.49	0.12	−0.84	−2.10
fish (ryba)	4.87	1	0.84	0.04	−0.75	0.99
grass (tráva)	4.79	2	0.01	1.53	−0.74	0.20
chair (stolička)	4.80	3	0.83	0.71	−0.71	1.19
warehouse (sklad)	4.61	2	−1.29	−0.16	−0.65	0.46
present (darček)	4.78	1	−0.65	1.07	−0.60	2.70
car (auto)	5.68	1	1.34	−0.53	−0.55	−1.18
shoe (topánka)	4.65	1	1.06	−0.18	−0.46	−0.12
sugar (cukor)	4.76	2	0.93	−0.11	−0.39	−0.32
drink (nápoj)	4.71	1	−1.03	1.45	−0.21	1.08
cable (kábel)	4.19	2	0.32	−0.67	−0.09	−0.11
window (okno)	5.24	3	0.30	0.55	0.00	0.55
chateau/lock (zámok)	5.18	2	−0.48	0.51	0.06	−1.09
kitchen/cuisine (kuchyňa)	4.96	3	−0.11	0.89	0.11	−0.42
card (karta)	5.24	3	−0.24	0.11	0.14	−1.57
root (koreň)	4.72	4	−1.46	0.51	0.25	−0.36
ear (ucho)	4.92	3	0.83	−0.30	0.31	−0.58
wheel (koleso)	4.64	3	0.73	−0.50	0.41	0.61
engine (motor)	4.90	3	−0.07	−0.81	0.50	−0.12
snow (sneh)	4.97	2	0.45	0.78	0.63	1.40
bottle (fľaša)	4.78	1	1.02	−0.72	0.64	0.70
bag (taška)	4.53	2	0.27	0.02	0.65	−0.71
sleeve (rukáv)	4.28	1	−0.01	−0.30	0.74	0.66
pub (krčma)	4.52	1	0.66	−1.63	0.80	−0.77
fuel (palivo)	4.54	1	−1.99	0.00	0.85	0.67
boat (čln)	4.48	1	0.76	−0.24	0.86	−0.71
tunnel (tunel)	4.51	2	0.07	−0.77	0.92	−1.65
cheese (syr)	4.59	1	0.65	0.48	0.99	−1.21
bank (banka)	5.68	3	−0.32	−0.85	0.99	−0.70
meadow (lúka)	4.61	1	−0.56	2.28	1.41	−0.90
universe (vesmír)	4.49	1	−0.81	0.60	1.45	−0.07
bee (včela)	4.28	1	1.51	−0.76	1.83	−0.60
palm (dlaň)	4.72	1	0.39	0.39	2.30	−1.49

**Table 6.** List of ADT word stimuli (form B). (Note: frequency represents logarithm of word frequency in the national corpus database and polysemy indicates the number of distinct meanings of the word found in the lexicon. Vividness, affective tone (high = positive and calm; low = negative and arousing), associative typicality, and associative topology (high = efficient; low = inefficient) are expressed in composite *z*-scores obtained from principal component analyses.)

stimulus word (SK)	frequency	polysemy	vividness	affective tone	associative typicality	associative topology
flower (kvet)	4.92	1	0.20	1.91	−1.41	−0.44
stair/step (schod)	4.73	1	0.63	−0.70	−1.35	0.27
dog (pes)	5.19	1	0.38	0.65	−1.24	0.68
brain (mozog)	4.79	2	0.57	−0.22	−1.15	0.25
picture (obrázok)	4.79	2	−2.18	1.32	−1.11	1.40
pillar (pilier)	4.71	1	−2.61	0.77	−1.06	−0.14
dust (prach)	4.65	2	−1.06	−1.38	−0.92	−0.50
beer (pivo)	4.90	1	1.13	0.10	−0.92	1.08
iron (železo)	4.28	2	−1.50	−0.44	−0.83	−2.72
top/hill (vrch)	4.80	1	−1.62	0.74	−0.65	0.74
bird (vták)	4.72	2	0.62	0.02	−0.57	0.33
barrel (barel)	4.50	2	−3.26	0.07	−0.53	−0.79
church (kostol)	5.25	1	0.68	−0.21	−0.51	−0.15
gate (brána)	5.28	3	−0.30	−0.02	−0.33	−0.78
radio (rádio)	4.95	2	−0.22	0.05	−0.33	0.68
bar (bar)	4.64	3	−0.38	−0.24	−0.23	1.29
fruit (ovocie)	4.81	1	−0.04	1.60	−0.18	−0.46
rock (skala)	4.65	2	−0.15	−0.05	−0.08	−0.18
jury (porota)	4.71	2	−2.30	−1.26	0.00	−0.18
statue (socha)	4.80	1	−0.81	0.68	0.01	−0.84
stage (pódium)	4.61	1	−0.29	−1.26	0.04	−0.77
wood (drevo)	4.94	2	−0.24	0.71	0.05	0.08
heart (srdce)	5.53	5	−0.27	0.81	0.10	1.21
fire (oheň)	4.93	3	0.78	−1.07	0.15	0.16
bear (medveď)	4.63	2	1.89	−2.44	0.53	0.34
smoke (dym)	4.50	1	−0.66	−1.50	0.57	0.77
package (balík)	4.77	3	−0.39	0.10	0.57	1.44
pupil (žiak)	5.54	3	−0.41	−0.24	0.81	0.42
bin (kôš)	4.63	4	0.51	−1.09	0.97	−0.88
ball (lopta)	5.20	1	1.48	−0.21	1.12	0.65
glasses (okuliare)	4.50	2	1.46	−0.37	1.16	−0.88
rail (koľaj)	4.19	3	0.81	−0.93	1.18	−0.08
lake (jazero)	4.71	1	0.14	1.62	1.19	−1.11
post office/mail (pošta)	4.90	3	−0.95	−0.09	1.29	−0.04
chimney (komín)	4.27	1	0.58	−0.93	1.36	−0.19
marble/bullet (guľka)	4.18	2	−0.61	−1.87	1.49	−2.37
mirror (zrkadlo)	4.69	3	0.58	−0.06	1.57	−1.47
beach (pláž)	4.49	1	−0.37	2.71	1.58	−0.06
thumb (palec)	4.48	2	0.96	−0.36	1.90	−0.25
lamp (lampa)	4.48	1	0.77	0.22	2.15	−0.77

**Table 7.** List of ADT word stimuli (form C). (Note: frequency represents logarithm of word frequency in the national corpus database and polysemy indicates the number of distinct meanings of the word found in the lexicon. Vividness, affective tone (high = positive and calm; low = negative and arousing), associative typicality, and associative topology (high = efficient; low = inefficient) are expressed in composite *z*-scores obtained from principal component analyses.)

stimulus word (SK)	frequency	polysemy	vividness	affective tone	associative typicality	associative topology
bathrobe/mayor (župan)	4.53	2	0.36	0.55	−1.92	1.80
oil (olej)	4.81	2	0.55	−0.56	−1.86	1.94
palace (palác)	4.79	1	−1.26	0.82	−1.52	−0.84
doctor/physician (doktor)	4.89	2	0.22	−1.14	−1.51	0.71
map (mapa)	4.72	2	−0.48	0.54	−1.49	−0.66
shirt (tričko)	4.57	2	1.18	−0.30	−1.44	1.03
tent (stan)	4.53	2	0.83	−0.10	−1.39	0.42
salt (soľ)	4.68	2	0.66	−0.21	−1.17	1.39
thief (zlodej)	4.69	1	−0.14	−3.58	−1.15	2.14
scarf (šatka)	4.20	1	−0.05	0.00	−1.09	0.90
wall (stena)	4.79	3	−0.14	−0.68	−1.08	0.12
concrete (betón)	4.21	3	−0.36	−0.63	−1.05	0.22
mansion (kaštieľ)	4.60	1	−0.70	0.45	−1.02	−2.74
soup (polievka)	4.59	1	0.81	0.90	−0.94	0.31
camera (kamera)	4.81	2	0.90	−0.96	−0.83	0.17
hill/bunch (kopa)	4.73	2	−0.29	0.23	−0.74	0.64
pipe/oven (rúra)	4.30	3	−0.39	0.01	−0.62	−1.06
dress (šaty)	4.88	2	0.81	0.38	−0.50	−0.87
meat (mäso)	4.94	2	0.70	−0.37	−0.42	0.08
tower (veža)	4.74	2	−0.16	−0.10	−0.31	−1.64
drug (droga)	4.81	2	−1.84	−2.13	−0.22	−2.10
sheep (ovca)	4.50	2	0.59	0.37	−0.16	2.09
room (izba)	5.24	1	−1.64	1.73	−0.16	−1.00
star (hviezda)	5.17	3	−1.59	2.46	0.06	1.16
tree (strom)	5.20	1	0.62	1.68	0.18	−0.47
pocket (vrecko)	4.80	2	−0.65	0.05	0.25	−0.12
photo (fotka)	4.53	1	−0.60	0.44	0.33	0.69
desk/board (doska)	4.64	6	−0.85	−0.06	0.34	−1.25
sand (piesok)	4.57	1	0.78	0.52	0.50	0.38
ankle (členok)	4.28	1	0.89	−0.46	0.50	0.02
door (dvere)	5.49	1	1.20	−0.42	0.51	1.27
knee (koleno)	4.92	2	1.16	−0.55	0.53	0.13
eye (oko)	5.74	4	0.79	−0.15	0.70	−0.03
rose (ruža)	4.49	1	0.91	1.11	0.71	1.15
soldier (vojak)	5.24	1	0.88	−2.25	1.09	0.06
airport (letisko)	4.96	1	0.31	−0.38	1.28	−0.98
sky (obloha)	4.70	2	−1.49	2.41	1.68	0.78
gallery (galéria)	4.95	3	−2.87	1.93	1.85	0.77
garage (garáž)	4.49	1	0.73	−0.19	1.89	−0.91
puck/sprout (puk)	4.61	2	1.17	−0.80	2.58	−0.37
